# The association between Geriatric Nutritional Risk Index and KSD disease: results from National Health and Nutrition Examination Survey 2007–2018

**DOI:** 10.3389/fnut.2024.1430668

**Published:** 2024-11-06

**Authors:** Zhicheng Tang, Hongzheng Zhong, Qingqing Zhi, Yinqiu Chen, JiaHao Zhang, Zhibiao Li, Zechao Lu, Can Liu, Fucai Tang, Zhaohui He, Xuan Sun

**Affiliations:** ^1^Department of Urology, The Eighth Affiliated Hospital, Sun Yat-sen University, Shenzhen, Guangdong, China; ^2^School of Medicine, Sun Yat-sen University, Shenzhen, China

**Keywords:** Geriatric Nutritional Risk Index (GNRI), Kidney Stone Disease (KSD), National Health and Nutrition Examination Survey (NHANES), nutritional status, risk factors

## Abstract

**Background:**

Kidney stone disease (KSD) is a widespread problem in urology. But the associations between the Geriatric Nutritional Risk Index (GNRI), an important indicator for assessing the nutritional status of elderly hospitalized patients, and KSD are understudied.

**Objective:**

Utilizing data from the National Health and Nutrition Examination Survey (NHANES) spanning 2007–2018, our study analyzed the correlation between the GNRI and KSD prevalence at cross-sectional level. The aim of the study was to explore the association between GNRI and the prevalence of KSD to identify potential risk factors and inform prevention and management strategies for KSD.

**Methods:**

This cross-sectional study analyzed data focusing on 26,803 adults (20–80 years) after screening for complete data. It evaluated GNRI, a formula involving albumin, present, and ideal body weight, stratifying participants into quartiles. The primary outcome was the history of KSD, based on self-reports. Covariates included demographic, health, and lifestyle factors. Statistical analysis employed *t*-tests, ANOVA, Wilcoxon, and Kruskal-Wallis tests, with logistic regression modeling GNRI’s impact on KSD prevalence, assessing odds ratios and potential multicollinearity, and sensitivity analyses excluding individuals with low eGFR and adjusting cycle years.

**Results:**

Significant disparities are found in GNRI distributions between individuals with and without kidney stones. Higher GNRI levels are more common in kidney stone patients, with 39.249% in the highest quartile versus 33.334% in those without stones. Notably, those in the highest GNRI quartile (Q4) show a lower prevalence of kidney stone disease (KSD) than those in the lowest (Q1), with rates of 11.988% versus 8.631%, respectively (*P* < 0.0001). Adjusted model results reveal that higher GNRI quartiles (Q3-Q4) correlate with reduced KSD prevalence, with odds ratios of 0.85 (95% CI [0.72, 1.00]) and 0.76 (95% CI [0.65, 0.89]). A nonlinear inverse relationship exists between GNRI levels and KSD prevalence across the population (*P* < 0.001), confirming that higher GNRI lowers KSD prevalence. Subgroup and sensitivity analyses support these findings.

**Conclusion:**

The study underscores a significant, albeit nonlinear, association between elevated GNRI levels and decreased KSD prevalence. This relationship highlights the importance of nutritional assessment and management in KSD prevention strategies.

## 1 Introduction

Kidney Stone Disease (KSD) represents a prevalent issue within urology, affecting approximately 11% of the population and boasting an prevalence rate of 14.8% (1, 2). This condition frequently results in renal colic, urinary tract infections, and kidney damage. Advances in diagnostic and surgical techniques, including Percutaneous Nephrolithotomy (PNL), Retrograde Intrarenal Surgery (RIRS), and Extracorporeal Shock Wave Lithotripsy (ESWL), have significantly enhanced the management of KSD. However, despite these advancements, the post-surgery recurrence rate of KSD remains alarmingly high, with up to 50% of patients experiencing recurrence within 5 years (2). This high recurrence rate underscores the necessity of investigating KSD formation’s risk factors and devising strategies to mitigate the risk of new or recurring instances, which is crucial for alleviating the considerable health and economic impacts associated with this condition.

Since its inception by Bouillanne et al. in 2005, the Geriatric Nutritional Risk Index (GNRI) has become a pivotal instrument for evaluating the nutritional status of hospitalized elderly patients, proving effective in predicting morbidity and mortality risks (3). Its simplicity and efficacy have broadened its application across various medical fields, including digestive system diseases, orthopedics, and urology (4–6). Grinstead et al. emphasize the importance of GNRI in monitoring the nutritional status to enhance cancer patients’ survival rates (7). It is documented that GNRI is a prognostic tool, which can predict severe complications and mortality of elderly patients undergoing colorectal cancer surgery (8). Further, Haas et al. have proposed GNRI as a potential predictor of immunotherapy response in patients with squamous cell carcinoma of the head and neck (9). Additionally, epidemiological studies identify GNRI as an independent risk factor for older people with osteoporosis, linking it to the condition’s severity (10). In the context of urologic diseases, Nakagawa et al. highlighted GNRI’s association with all-cause and cardiovascular mortality (11). Miao et al. found an inverse correlation between GNRI and prostate cancer risk, suggesting a nonlinear relationship (12). Shu et al. demonstrated GNRI’s predictive value for postoperative outcomes in prostate cancer surgery (13). Despite the known correlation between nutritional factors and KSD formation (14), the specific relationship between GNRI and KSD prevalence warrants further investigation.

The National Health and Nutrition Examination Survey (NHANES) is a comprehensive, cross-sectional survey that collects health and nutrition data from households across the United States. Through stratified multistage sampling, NHANES ensures its sample is representative of the U.S. population, covering demographic, socioeconomic, dietary, and health data obtained through household interviews and health examinations. Utilizing this rich database, our study conducted a retrospective analysis to explore the association between the Geriatric Nutritional Risk Index (GNRI) and the prevalence of kidney stone disease (KSD). This investigation aims to deepen our understanding of the risk factors associated with KSD, offering new insights for its prevention and management.

## 2 Materials and methods

### 2.1 Data source and study population

**FIGURE 1:**
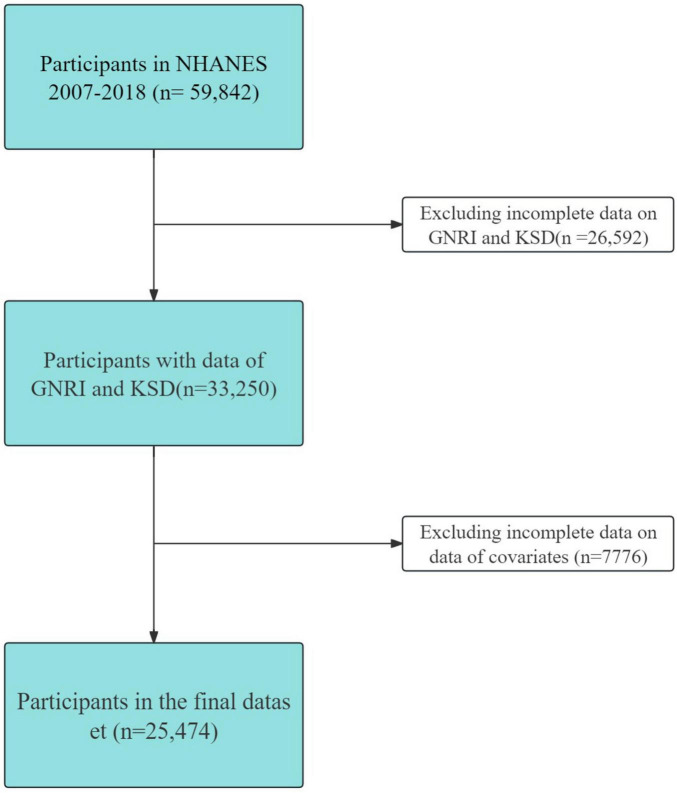
Inclusion and exclusion criteria. NHANES, National Health and Nutrition Examination Survey.

This cross-sectional study utilized data from the National Health and Nutrition Examination Survey (NHANES), a resource managed by the National Center for Health Statistics at the Centers for Disease Control and Prevention. The NHANES website’s publicly available data plays a crucial role in epidemiological research and in assessing nutritional health. Our research focused on the 2007–2018 NHANES dataset, encompassing participants aged 20–80 years and providing comprehensive, reliable information across various domains consisting of demographics, dietary and health-related behaviors, physical measurements, and disease information. The initial participant pool of 59,842 was refined to 25,474 eligible subjects for this study. The refinement process included screening for completeness in dataset outcomes and exposures (*n* = 26,592), and relevant covariates (*n* = 7776). These covariates encompassed annual household income (1896), marital status (14), education level (29), eGFR (1856), BMI (364), smoking status (13), alcohol use (3220), sitting time (113), cardiovascular disease (1), hypertension (1), diabetes mellitus (269), and moderate recreational activity (0), as illustrated in Figure 1.

### 2.2 Assessment of GNRI

The GNRI is recognized as a straightforward yet effective score for nutritional assessment. The GNRI formula incorporates serum albumin levels (expressed in g/L), present body weight (PBW, in kg), and ideal body weight (IBW), where IBW is calculated as the square of height in meters (m^2^) multiplied by 22. The GNRI is calculated using the following equation: GNRI = 1.489 × albumin ++ 41.7 × (PBW/IBW). In cases where PBW surpasses IBW, the PBW/IBW ratio is set to 1. For our study, participants were stratified into four equal groups based on their GNRI scores: Q1 (61.96–101.26), Q2 (101.26–104.24), Q3 (104.24–107.22), and Q4 (107.22–125.08). This stratification facilitated a comprehensive analysis of nutritional risk across different levels.

### 2.3 Assessment of KSD

In the NHANES survey, the primary outcome assessed was the presence of a history of KSD disease, dichotomously categorized as “yes” or “no.” This information was garnered through a structured questionnaire survey. Participants affirmatively responding to the query “Have you ever had KSD?” were categorized as having a history of KSD disease. This method of classification was based on self-reported data (15).

### 2.4 Assessment of covariates

In this study, we meticulously adjusted for a broad range of confounding factors to clarify the relationship between the prevalence of KSD and the GNRI. The adjustment included sociodemographic characteristics, Body Mass Index (BMI, in kg/m^2^), history of chronic diseases, physical activity levels, cardiovascular diseases, glomerular filtration rate (eGFR), and cycle years. The sociodemographic variables comprised age, gender (male or female), race (White or other), marital status (unmarried or married), annual household income (below $20,000 or above $20,000), and educational level (below high school, high school graduate, or above high school). Chronic diseases considered were cardiovascular diseases, hypertension, and diabetes, with options for response as “no” or “yes”. Smoking and drinking habits were also included, categorized as “more” or “less” for smoking, and “no” or “yes” for drinking. BMI was classified into two categories: less than 25, and 25 or above. Physical activity assessment encompassed moderate recreational activities and sedentary time, with responses of “no” or “yes”. The occurrence of KSD was another covariate, with “no” or “yes” responses. The Chronic Kidney Disease Epidemiology Collaboration (CKD-EPI) equation (19414839) was used to estimate eGFR. Our sensitivity analyses involved considering eGFR as a covariate. The study spanned six biennial cycles from 2007-2008 to 2017-2018, providing a temporal framework for our analysis (16, 17).

### 2.5 Statistical analysis

This study delineates the presentation and examination of various data types. Continuous variables that show normal or near-normal distribution are described by mean values (standard error, SE), with the application of *t*-tests for two-group comparisons while one-way ANOVA for scenarios involving three or more groups. In contrast, Continuous variables that are not normally distributed are depicted as median values (interquartile range, IQR) while evaluated using Wilcoxon tests for two groups and Kruskal-Wallis tests for three or more groups. Categorical variables are represented as actual sample numbers (weighted percentages) while Chi-square tests are used to identify significant differences in distribution.

For the investigation of GNRI we categorized these variables into quartiles and implemented a weighted binary logistic regression model to examine its association with the prevalence of KSD. This approach facilitated the presentation of associations as odds ratios (OR) accompanied by 95% confidence intervals (95% CI). To address potential multicollinearity, we utilized variance inflation factors, with values exceeding 10 indicating significant multicollinearity. Dummy variables were introduced for the effective handling of categorical variables.

We also conducted subgroup analyses to evaluate interaction effects, using weighted likelihood ratio tests. The fully adjusted model was used to analyze multiplicative interaction terms for each covariate, except for the stratified covariate. To ensure the robustness of our findings, the study incorporated several sensitivity analyses. These included excluding individuals with an estimated glomerular filtration rate (eGFR) lower than 60 mL/min/1.73m^2^ and adjusting for cycle years.

R software, version 4.2.1 (R Core Team), was used for all statistical procedures. A 2-tailed *P*-value threshold of lower than 0.05 was applied to determine statistical significance.

## 3 Results

This study included 25,474 participants after excluding individuals whose information was incomplete and who had missing data on GNRI, KSD and covariates (Figure 1).

### 3.1 Characteristics of the study population according to the KSD

In the preliminary analysis (Table 1), significant differences were observed between the groups regarding variables including age, sex, race, marital status, cardiovascular disease, hypertension, diabetes mellitus, smoke, BMI, moderate recreational activity, GNRI.

**TABLE 1:** Characteristics of the study population according to the kidney stone disease (KSD).

Variable	Without KSD	With KSD	*P*-value
Age (*n*, %)			<0.0001
<65	17838 (82.591)	1639 (74.380)	
≥65	5176 (17.409)	821 (25.620)	
Sex (*n*, %)			<0.0001
Female	11747 (51.363)	1073 (44.781)	
Male	11267 (48.637)	1387 (55.219)	
Race (*n*, %)			<0.0001
Other	13307 (32.221)	1081 (21.709)	
White	9707 (67.779)	1379 (78.291)	
Marital status (*n*, %)			<0.0001
No	6293 (27.342)	387 (15.648)	
Yes	16721 (72.658)	2073 (84.352)	
Annual household income (*n*, %)			0.526
<20,000	4715 (13.461)	565 (13.925)	
>20,000	18299 (86.539)	1895 (86.075)	
Education level (*n*, %)			0.496
≤High school	10548 (37.438)	1152 (38.362)	
>High school	12466 (62.562)	1308 (61.638)	
Cardiovascular disease (*n*, %)			<0.0001
No	20721 (92.169)	1994 (85.008)	
Yes	2293 (7.831)	466 (14.992)	
Hypertension (*n*, %)			<0.0001
No	13503 (63.731)	1071 (47.333)	
Yes	9511 (36.269)	1389 (52.667)	
Diabetes mellitus (*n*, %)			<0.0001
No	18872 (86.727)	1723 (75.319)	
Yes	4142 (13.273)	737 (24.681)	
Smoke (*n*, %)			<0.0001
Less	12875 (56.229)	1201 (50.077)	
More	10139 (43.771)	1259 (49.923)	
Alcohol user (*n*, %)			0.971
No	3247 (10.644)	331 (10.609)	
Yes	19767 (89.356)	2129 (89.391)	
BMI (kg/m^2^, SE)	28.959 (0.088)	30.616 (0.161)	<0.0001
Moderate recreational activity (*n*, %)			<0.0001
No	11648 (44.219)	1445 (51.970)	
Yes	11366 (55.781)	1015 (48.030)	
Sitting time (*n*, %)			0.430
<5	9466(37.239)	973 (36.138)	
≥5	13548 (62.761)	1487(63.862)	
eGFR (mL/min/1.73 m2, SE)	94.883 (0.350)	87.772 (0.529)	<0.0001
Geriatric Nutritional Risk Index (*n*, %)			<0.0001
Q1	6531 (25.044)	813 (30.668)	
Q2	2797 (11.177)	281 (11.355)	
Q3	5613 (24.531)	609 (24.643)	
Q4	8073 (39.249)	757 (33.334)	

The prevalence of KSD in the group aged over 65 was significantly above the prevalence in the group aged under 65. Also, the KSD group had a wider proportion of males and white people than the without KSD group.

With regard to social factors, the KSD group exhibited a larger percentage of married individuals, but there was no noteworthy discrepancy with respect to annual household income and educational level. Otherwise, the KSD group had a higher proportion of individuals with regards to cardiovascular disease, hypertension, diabetes mellitus, more smoking and moderate recreational activity (*P* < 0.0001) as well as the higher BMI score and lower eGFR (*P* < 0.001). Moreover, alcohol user and sitting time were not statistically different (*P* > 0.05; Table 1). Overall, the KSD group tends to have a higher proportion of low GNRI scores.

### 3.2 Characteristics of the study population according to GNRI

Table 2 present the cohort characteristics distribution categorized according to GNRI level. In the preliminary analysis, as the GNRI score increases, the proportion of people aged over 65 years decreases. Regarding social factors, the proportion of people who are males, white, annual household income > 20,000, education level > High School, alcohol users as well as having moderate recreational activity increased while the GNRI increased from Q1 to Q4. However, as for the systemic disease factors, as the GNRI score increases, the proportion of people with cardiovascular disease, hypertension, diabetes mellitus went on a decreased trend (*P* < 0.05). Interestingly, while the GNRI increased, the BMI of participants dropped but the eGFR of participants elevated. Moreover, the prevalence of KSD among the participants decreased from 11.988 to 8.631% while the GNRI increased, (*P* < 0.0001).

**TABLE 2:** Characteristics of the study population according to GNRI.

Variable	GNRI Q1	GNRI Q2	GNRI Q3	GNRI Q4	*P*-value
Age (*n*, %)					<0.0001
<65	5178 (75.870)	2261 (77.611)	4703 (81.089)	7335 (87.313)	
≥65	2166 (24.130)	817 (22.389)	1519 (18.911)	1495 (12.687)	
Sex (*n*, %)					<0.0001
Female	4915 (70.256)	1842 (62.469)	3074 (50.462)	2989 (34.500)	
Male	2429 (29.744)	1236 (37.531)	3148 (49.538)	5841 (65.500)	
Race (n, %)					<0.0001
Other	4416 (35.545)	1793 (33.904)	3482 (29.869)	4697 (28.304)	
White	2928 (64.455)	1285 (66.096)	2740 (70.131)	4133 (71.696)	
Marital status (n, %)					<0.0001
No	1773 (23.856)	755(24.669)	1502 (23.469)	2650 (29.857)	
Yes	5571 (76.144)	2323 (75.331)	4720 (76.531)	6180 (70.143)	
Annual household income (*n*, %)					<0.0001
<20,000	1840 (17.891)	659 (15.217)	1249 (12.323)	1532 (10.862)	
>20,000	5504 (82.109)	2419 (84.783)	4973 (87.677)	7298 (89.138)	
Education level (*n*, %)					<0.0001
≤High school	3599 (41.672)	1404 (37.438)	2849 (37.222)	3848 (35.010)	
>High school	3745 (58.328)	1674 (62.562)	3373 (62.778)	4982 (64.990)	
Cardiovascular disease (*n*, %)					<0.0001
No	6216 (87.140)	2729 (89.338)	5609 (92.494)	8161 (94.260)	
Yes	1128 (12.860)	349 (10.662)	613 (7.506)	669 (5.740)	
Hypertension (*n*, %)					<0.0001
No	3689 (55.809)	1725 (61.322)	3553 (61.713)	5607 (66.711)	
Yes	3655(44.191)	1353(38.678)	2669(38.287)	3223(33.289)	
Diabetes mellitus (*n*, %)					<0.0001
No	5462 (79.336)	2457 (85.007)	5078 (85.639)	7598 (89.858)	
Yes	1882 (20.664)	621 (14.993)	1144 (14.361)	1232 (10.142)	
Smoke (*n*, %)					0.377
Less	3988 (55.728)	1705 (53.777)	3442 (55.379)	4941 (56.217)	
More	3356 (44.272)	1373 (46.223)	2780 (44.621)	3889 (43.783)	
Alcohol user (*n*, %)					<0.0001
No	1204 (13.043)	503 (12.771)	874 (10.446)	997 (8.555)	
Yes	6140 (86.957)	2575 (87.229)	5348 (89.554)	7833 (91.445)	
BMI (kg/m2, SE)	31.291 (0.157)	29.056 (0.204)	29.008 (0.126)	27.784 (0.088)	<0.0001
Moderate recreational activity (*n*, %)					<0.0001
No	4433 (55.235)	1648 (47.175)	3190 (45.100)	3822 (37.514)	
Yes	2911 (44.765)	1430 (52.825)	3032 (54.900)	5008 (62.486)	
Sitting time (*n*, %)					0.269
<5	2894 (37.409)	1289 (39.041)	2557 (37.220)	3699 (36.331)	
≥5	4450 (62.591)	1789 (60.959)	3665 (62.780)	5131 (63.669)	
eGFR (mL/min/1.73 m2, SE)	91.935 (0.536)	93.633 (0.620)	93.731 (0.451)	96.088 (0.433)	<0.0001
Kidney Stone Disease (*n*, %)					<0.0001
No	6531 (88.012)	2797 (89.848)	5613 (89.950)	8073 (91.369)	
Yes	813 (11.988)	281 (10.152)	609 (10.050)	757 (8.631)	

### 3.3 Associations between PTN and KSD based on the outcome for KSD status

Taking Quartile 1 as a reference for GNRI, a multifactorial logistic regression analysis was conducted to investigate the potential association between high-GNRI and the prevalence of KSD (Table 3). In the relationship equation, height and weight parameters are crucial for calculating Body Mass Index (BMI) and similarly critical in the GNRI formula. Considering this, we have made suitable adjustments to all analysis models to control for potential confounding factors.

**TABLE 3:** Associations between GNRI and KSD based on the outcome for KSD status.

Character	Model 1 OR (95%CI)	Model 2 OR (95%CI)	Model 3 OR (95%CI)
**GNRI**			
Q1	Reference	Reference	Reference
Q2	0.89 (0.72, 1.10)	0.87 (0.70, 1.07)	0.89 (0.72, 1.09)
Q3	0.89 (0.76, 1.03)	0.82 (0.70, 0.97)*	0.85 (0.72, 1.00)*
Q4	0.78 (0.68, 0.89)***	0.73 (0.63, 0.85)***	0.76 (0.65, 0.89)***
P for trend	**<0.001**	**<0.0001**	**<0.001**

^a^Model outcome was KSD (binary: without KSD and with KSD).

***p < 0.001 and *p < 0.05. ^b^Model 1: adjusted for BMI. ^c^Model 2: adjusted for BMI, age, sex, ethnicity, marital status, annual household income, education, smoked status, alcohol use, recreational activity and sitting time. ^d^Model 3: adjusted for BMI, age, sex, ethnicity, marital status, annual household income, education, smoked status, alcohol use, recreational activity, sitting time and cardiovascular disease, hypertension, diabetes mellitus. Bold text indicates statistical significance (*p* ≤ 0.05).

Model 1 has no adjustment for variables apart from BMI. Quartile 2 and 3 for GNRI showed no association with the prevalence of KSD. However, quartile 4 (OR = 0.78, 95% CI 0.68,0.89) demonstrated a decreased prevalence of KSD.

In addition, both adjusted Model 2 and 3 revealed that quartile 3 and 4 of GNRI was negatively associated with the prevalence of KSD. After adjusting for age, sex, ethnicity, marital status, annual household income, education, smoked status, alcohol use, recreational activity, sitting time and BMI, cardiovascular disease, hypertension, diabetes mellitus, it was found that in the fully adjusted model 3, [Q3, OR and 95% CI: 0.85(0.72,1.00); Q4, OR and 95% CI: 0.76(0.65,0.89)] was shown to have a more potent relationship that elevated GNRI linked to a lower prevalence of KSD (*P* < 0.0001).

### 3.4 Nonlinear association between GNRI and predicted KSD

**FIGURE 2:**
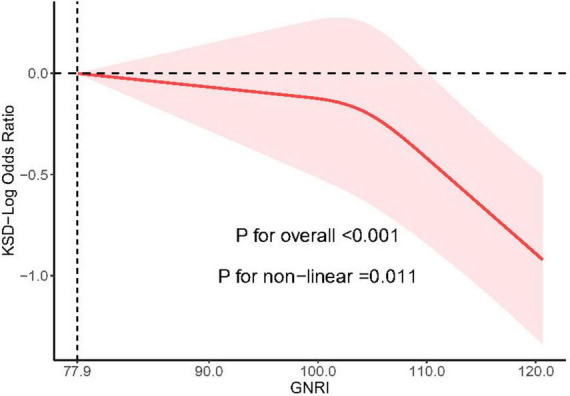
Restricted cubic spline for GNRI and prevalence of KSD based on the fully adjusted model.

A substantial non-linear association between the prevalence of KSD and GNRI in the NHANES cohort was confirmed by restricted cubic spline analysis, obtained by analyzing the 3 knots selected with the lowest AIC score (*P* = 0.001). As GNRI increased over 77.9, the overall prevalence of KSD dropped steadily (*P* < 0.05) (*P* < 0.05) (Figure 2).

### 3.5 Subgroup analysis

The interactive effect between GNRI and KSD was examined in our subgroup analyses using restricted cubic spline models (Supplementary Figures 1–3). The results indicate a nonlinear negative association between GNRI and the prevalence of KSD in subgroups including those younger than 65 years, males, white individuals, married participants, and those with higher economic status, higher education levels, hypertension, alcohol use, moderate recreational activity, and sedentary time ≥5 h (*P* < 0.05).

Conversely, no nonlinear correlation was observed between GNRI and KSD prevalence in subgroups characterized by lower education levels, absence of cardiovascular disease, hypertension, or diabetes, and varying smoking and drinking habits, as well as lack of moderate recreational activities. However, a negative linear correlation was present in these subgroups (*P* < 0.05). Notably, in the diabetes subgroup, a positive association with KSD prevalence was detected when GNRI was below 102.7.

### 3.6 Sensitivity analysis

On the basis of the original Model 3, two sensitivity analyses were conducted to validate the robustness of the results. These analyses consistently indicated that the relationship between KSD prevalence and the GNRI was significant, as detailed in Supplementary Tables 1, 2. The first sensitivity analysis excluded participants with an estimated glomerular filtration rate (eGFR) less than 60 mL/min, with results shown in Supplementary Table 1. The second analysis accounted for annual variations in KSD risk by adjusting for NHANES cycle years, detailed in Supplementary Table 2. Across these sensitivity analyses, the initial association found between GNRI and KSD in this study remained consistent and unaltered.

## 4 Discussion

Our large-scale sample study based on the NHANES database found that an elevation in the GNRI is associated with an increased prevalence of KSD, revealing a significant nonlinear relationship between the two. Our finding emphasizes the complexity of the interaction between nutritional status and the prevalence of KSD, indicating that lower GNRI values—typically indicative of inferior nutritional status—could, in a paradoxical twist, augment the prevalence of KSD formation under specific circumstances.

Current research underscores the significant correlation between metabolic and nutritional factors and the prevalence of KSD formation. A comprehensive study by Chang et al., involving over 121,000 participants, highlighted that metabolic syndrome components, such as hypertension, obesity, hyperlipidemia, and hyperglycemia, markedly elevate KSD risk (18). Nutritional factors, vital for metabolic processes by providing substrates, have garnered attention in KSD research. Investigations into dietary impacts reveal that potassium and calcium-rich foods, alongside a balanced vegetarian and dairy diet, as suggested by researchers like Chewcharat and Ferraro, could offer protective benefits against KSD (19, 20). Liu et al.’s work on the dietary inflammation index further established a positive link with KSD prevalence (21). Contrarily, antioxidant content in diets appears to have a negligible impact on stone formation rates (22, 23). The intersection of nutrition and metabolism has prompted a focus on physiological indicators, such as obesity-related measures like waist circumference, as potential predictors for KSD (24). Moreover, some studies have examined dietary patterns using 28 food parameters, such as proteins, carbohydrates, and fiber, to assess participants’ dietary inflammatory index. These studies found a positive correlation between higher dietary inflammatory index scores and an increased prevalence of kidney stones, underscoring the significant influence of dietary patterns on kidney stone prevalence (21). Despite these efforts, there remains a scarcity of research exploring the direct link between individual nutritional status and kidney stone risk. To address this gap, our study utilizes the Geriatric Nutritional Risk Index (GNRI), which integrates serum albumin and body weight, to investigate how individual nutritional levels might correlate with kidney stone risk.

As one of the indices included in the Geriatric Nutritional Risk Index (GNRI), the association between body weight and kidney stones has been extensively studied (25). Excessive body weight is widely considered one of the risk factors for the formation of kidney stones. Kemal-Sarica et al. note that being overweight is a substantial risk factor for the formation of kidney stones, and another meta-analysis confirms that being overweight significantly increases the risk of kidney stone recurrence (26, 27). A meta-analysis of observational studies conducted from 2005 to 2022 found that obese individuals had a higher odds ratio (OR) for kidney stones compared to non-obese individuals, at 1.35 (95% CI 1.20–1.52, *P* < 0.001) (28). Additionally, studies by Lee et al. have established Body Mass Index (BMI) as a significant risk factor for KSD, attributing to obesity, abdominal obesity, and abnormal fat distribution (24). Likewise, research by Zheng and his team involving more than 3000 participants demonstrated that body fat percentage is a dependable predictor of kidney stone disease, underscoring the strong connection between markers of obesity and kidney stone risk (29). However, it should be noted that in calculating the GNRI, if an individual’s actual weight exceeds their ideal weight, the GNRI score does not increase with increasing weight, which may limit its responsiveness to levels of overweight.

Serum albumin levels are a critical component of the Geriatric Nutritional Risk Index (GNRI) scoring, influencing the total score positively. Our research indicates a potential association between higher serum albumin levels and a reduced prevalence of kidney stones, though the direct relationship remains somewhat elusive. Serum albumin, known for its ability to bind calcium, plays a significant role in calcium homeostasis (30). When albumin levels decrease, there is a reduction in calcium ions bound to albumin, leading to an increase in free serum calcium (31). This condition can elevate urinary calcium excretion in the kidneys, thereby promoting stone formation (32). During the acute inflammatory phase, serum albumin levels are inversely correlated with systemic inflammation, which is supported by findings that hypoalbuminemia is associated with elevated inflammatory markers (33, 34). These markers are positively correlated with the prevalence of kidney stones, suggesting an indirect link between low albumin levels and stone formation (35). Additionally, albumin serves as a vital non-enzymatic antioxidant with free radical scavenging properties, playing a central role in maintaining plasma redox status and influencing oxidative stress levels, which are factors in kidney stone formation (36, 37). As the most abundant protein in human plasma, albumin’s roles extend beyond metabolic functions to include acting as a carrier for hormones, sterols, fatty acids, and drugs, and it is involved in antioxidant activity, immune regulation, and the inflammatory response (38). High serum albumin levels generally reflect good nutritional status and may correlate with higher socioeconomic status, where better access to healthcare and proactive disease prevention could contribute to lower prevalence of kidney stone disease.

This study utilizes a large and representative dataset from the National Health and Nutrition Examination Survey (NHANES), ensuring a broad and diverse sample that enhances the generalizability of the findings. Through an in-depth analysis of the relationship between the Geriatric Nutritional Risk Index (GNRI) and the prevalence of kidney stones, the research provides detailed insights into this association. Advanced statistical methods, specifically complex weighted multivariable logistic regression analysis, allow for robust adjustment of various confounding variables, thereby strengthening the validity of the results. Furthermore, conducting sensitivity analyses in both the general population and specific subgroups increases the reliability and robustness of the findings, ensuring they are applicable across different segments of the population. The study’s significant findings reveal a nonlinear relationship between increased GNRI levels and a reduced prevalence of kidney stones. This contributes valuable knowledge to the field and underscores the potential importance of nutritional risk assessment in the prevention of kidney stones.

Despite the strengths of this study, it is not without limitations. Firstly, while the use of NHANES data ensures a broad representation, it may still carry inherent limitations that could restrict the general applicability of our conclusions. Thus, our findings require external validation in other populations to confirm their robustness. Secondly, the cross-sectional design of the study precludes us from establishing causality. Moreover, despite the thorough design of the questionnaire, the potential for recall bias and variability in participants’ comprehension abilities could introduce further biases into the data. Lastly, the covariates adjusted in the model might not be true confounders, potentially affecting the observed associations. Moreover, the reliance on self-reported KSD could introduce recall bias and misclassification, further influencing the results. Lastly, additional epidemiological studies across diverse conditions and populations, as well as prospective cohort studies, are necessary to provide more comprehensive guidance for the clinical prevention of kidney stones.

In summary, this study’s exploration of the association between GNRI and KSD prevalence highlights the importance of weight management and maintaining serum albumin levels in the prevention of KSD. Using GNRI as a metric to assess individual KSD risk allows healthcare providers to identify those at higher risk due to nutritional or weight-related factors. Future research should focus on clarifying the direct influence of GNRI on KSD risk and investigating the potential of nutritional interventions in reducing this risk.

## 5 Conclusion

This study emphasizes the association between elevated GNRI levels and a reduced prevalence of KSD, revealing a significant nonlinear relationship between them. Such insights are crucial for formulating targeted prevention strategies for KSD, underscoring the importance of nutritional assessment and management in mitigating this risk.

## Data Availability

Publicly available datasets were analyzed in this study. This data can be found here: https://www.cdc.gov/nchs/nhanes/index.htm.
