# Supplementary material for: The association between Geriatric Nutritional Risk Index and KSD disease: results from National Health and Nutrition Examination Survey 2007–2018
Source: Front Nutr. 2024 Nov 6;11:1430668. doi: 10.3389/fnut.2024.1430668 (PMC11580257; doi:10.3389/fnut.2024.1430668)
Supplement: Supplementary file 1 [file Data_Sheet_1.ZIP › 07STable1-KSD-model adjusting -eGFR .docx]

**Table S1.** Associations between GNRI and KSD based on the outcome for KSD status after excluding eGFR＜60 ml/min.

| Character | Model 1  OR (95%CI) | Model 2  OR (95%CI) | Model 3  OR (95%CI) |
| --- | --- | --- | --- |
| GNRI | | | |
| Q1 | Reference | Reference | Reference |
| Q2 | 0.90(0.71,1.14) | 0.87(0.68,1.11) | 0.89(0.70,1.12) |
| Q3 | 0.86(0.72,1.03) | **0.79(0.66,0.95) **** | **0.81(0.67,0.97) *** |
| Q4 | **0.77(0.66,0.89) ***** | **0.70(0.59,0.83) ***** | **0.73(0.61,0.86) ***** |
| P for trend | **<0.001** | **<0.0001** | **<0.001** |

^a^ Model outcome was KSD (binary: without KSD and with KSD). ***p < 0.001 **p < 0.01 and *p <0.05.

^b^ Model 1: adjusted for BMI

^c^ Model 2: adjusted for BMI, age, sex, ethnicity, marital status, annual household income, education, smoked status, alcohol use, recreational activity and sitting time.

^d^ Model 3: adjusted for BMI, age, sex, ethnicity, marital status, annual household income, education, smoked status, alcohol use, recreational activity, sitting time and cardiovascular disease, hypertension, diabetes mellitus.
