# Supplementary figures and images for: The association between Geriatric Nutritional Risk Index and KSD disease: results from National Health and Nutrition Examination Survey 2007–2018
Source: Front Nutr. 2024 Nov 6;11:1430668. doi: 10.3389/fnut.2024.1430668 (PMC11580257; doi:10.3389/fnut.2024.1430668)

A

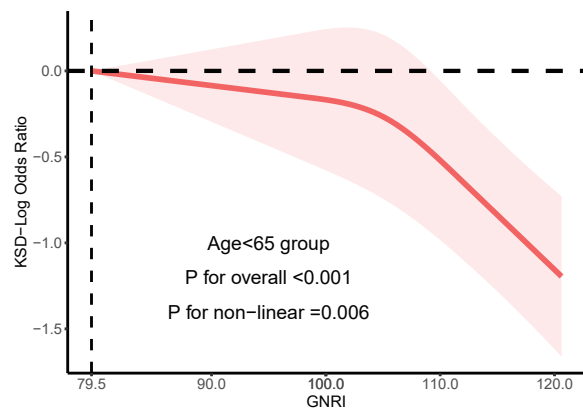

B

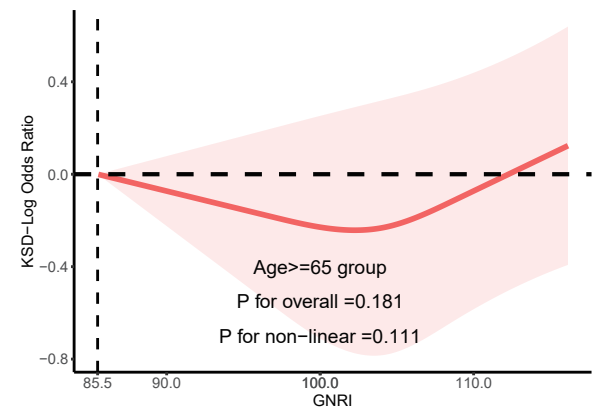

C

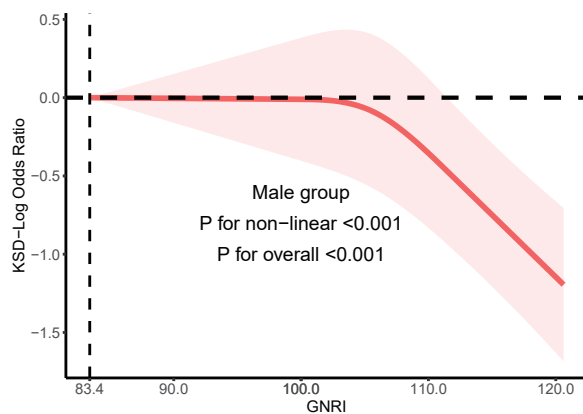

D

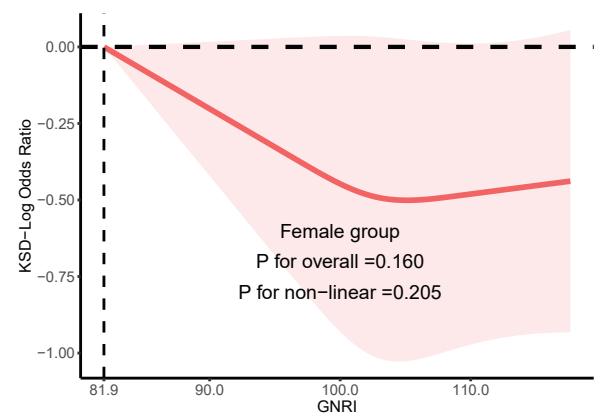

E

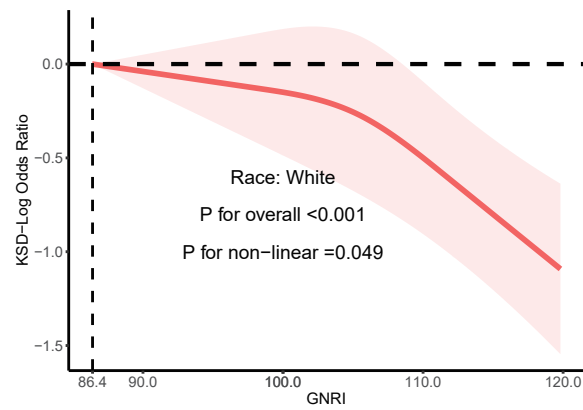

F

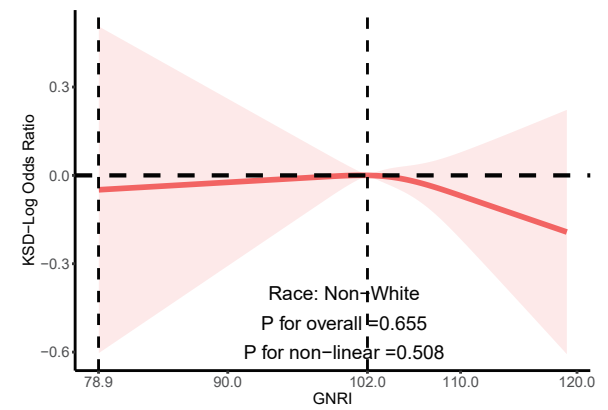

G

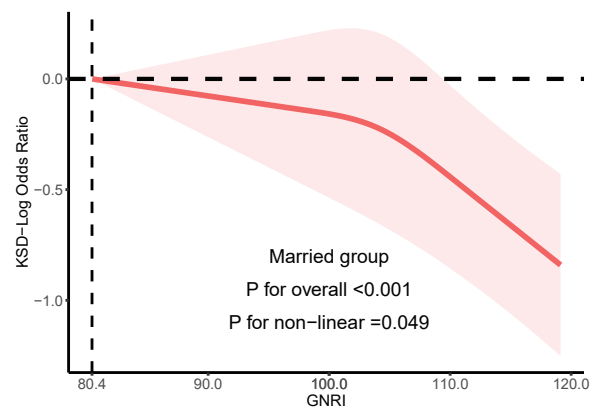

H

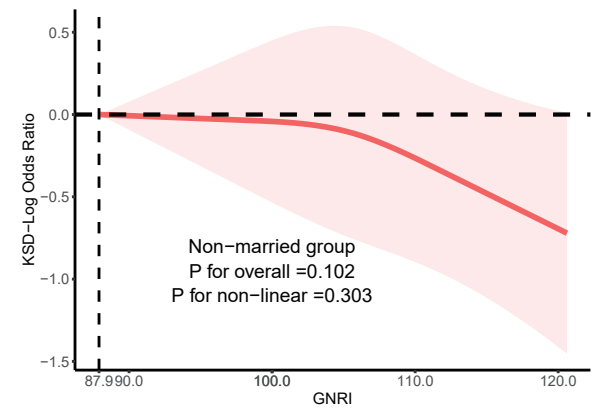

Supplement: Supplementary file 1 [file Data_Sheet_1.ZIP › Supplementary Figure1.pdf]

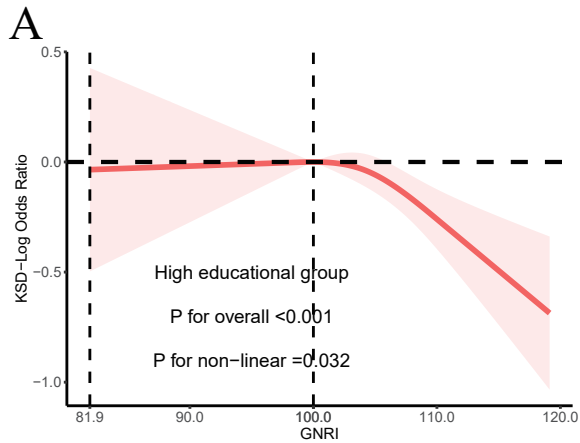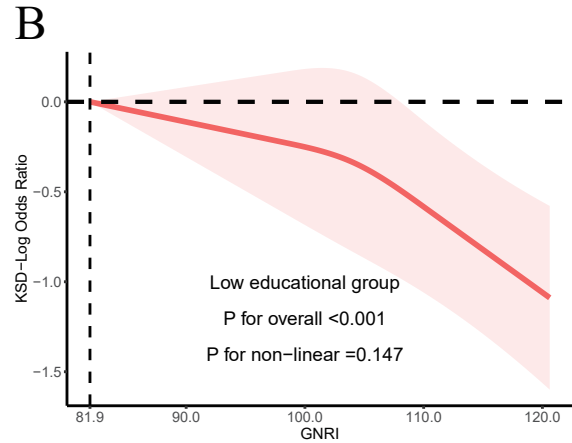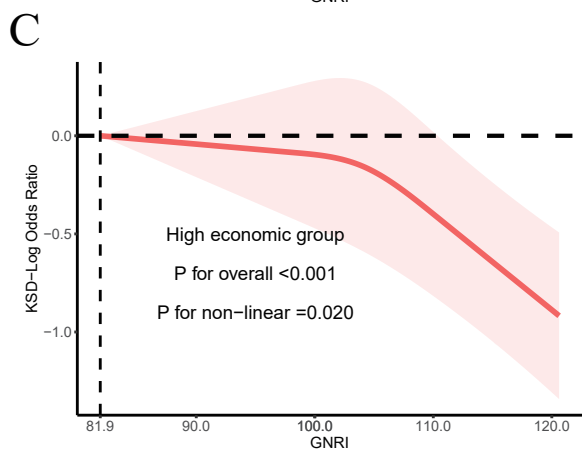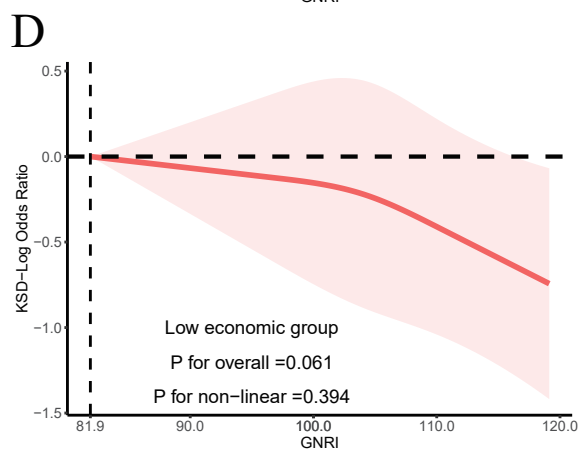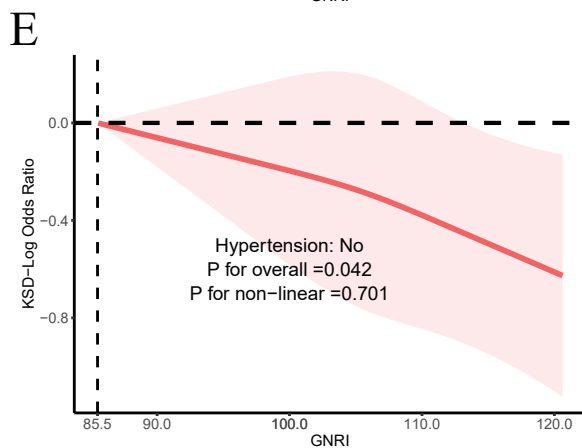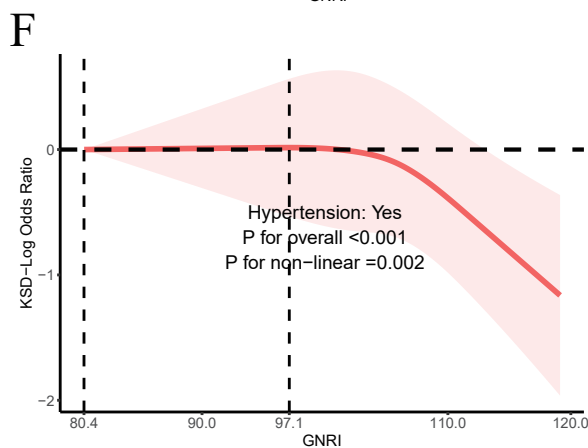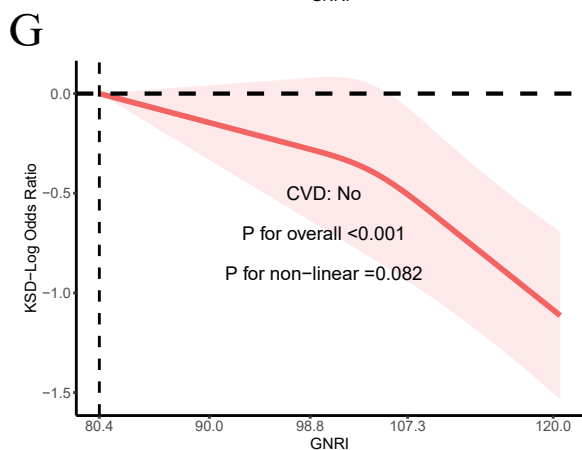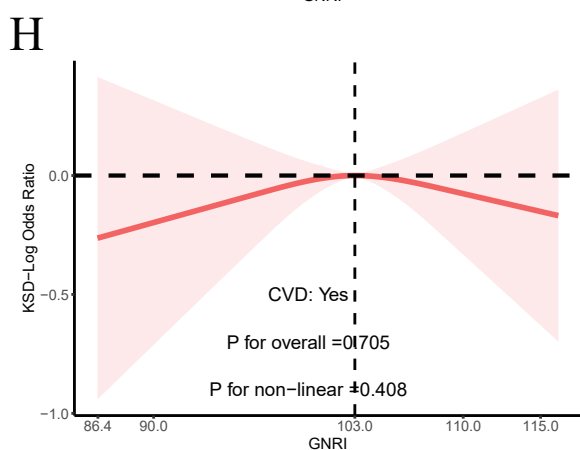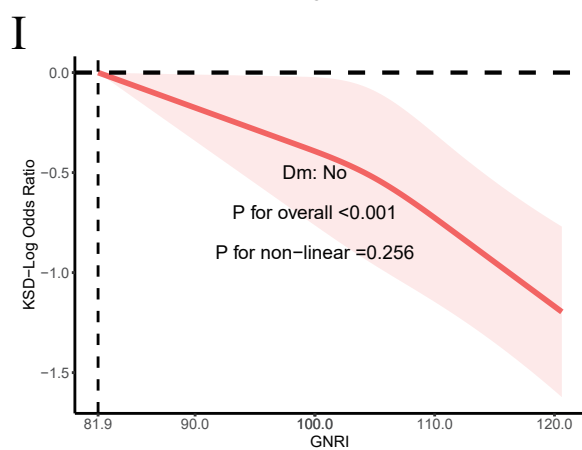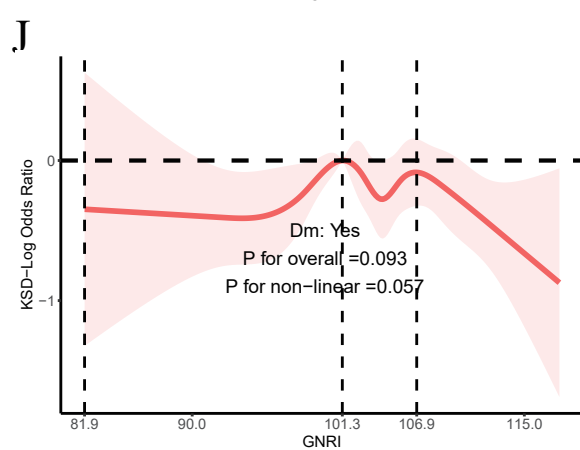

Supplement: Supplementary file 1 [file Data_Sheet_1.ZIP › Supplementary Figure2.pdf]

A

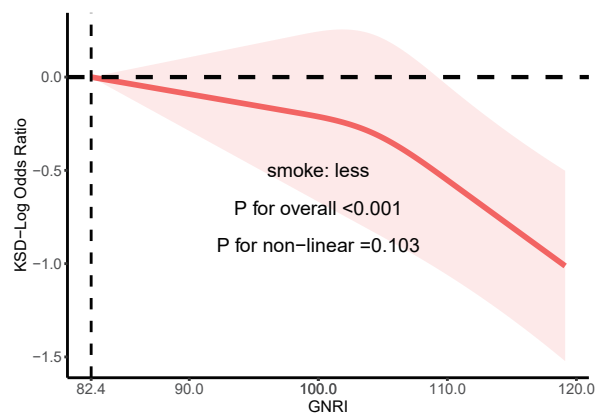

B

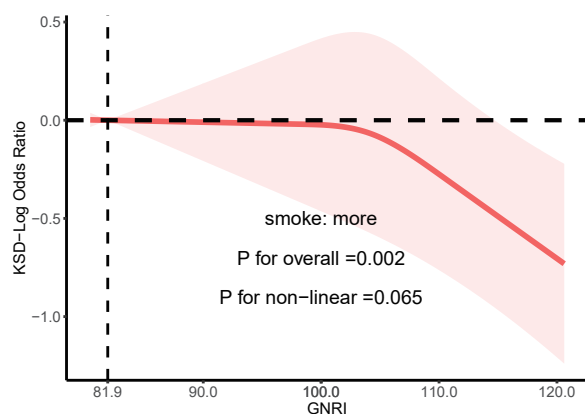

C

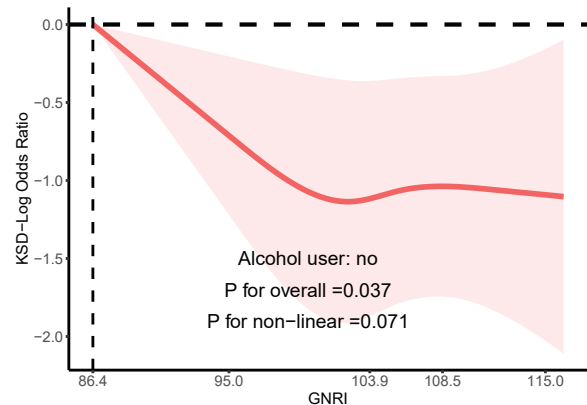

D

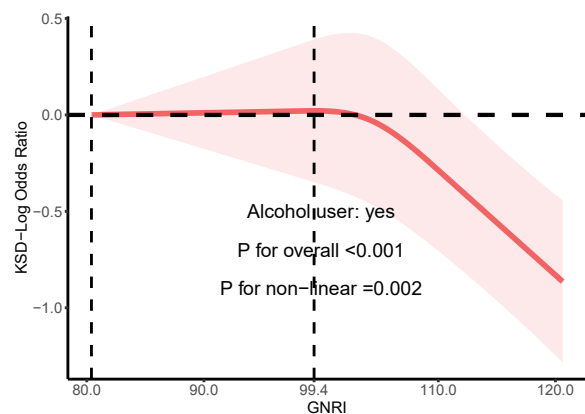

E

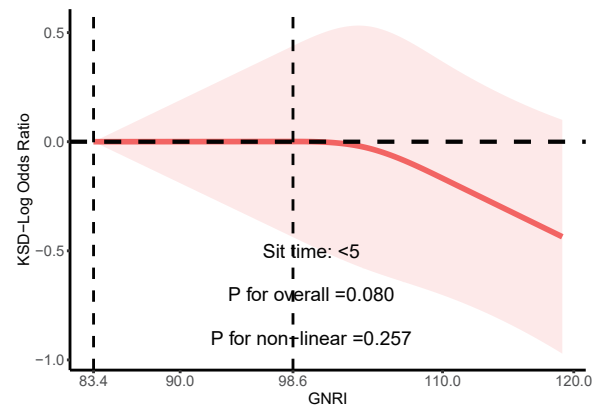

F

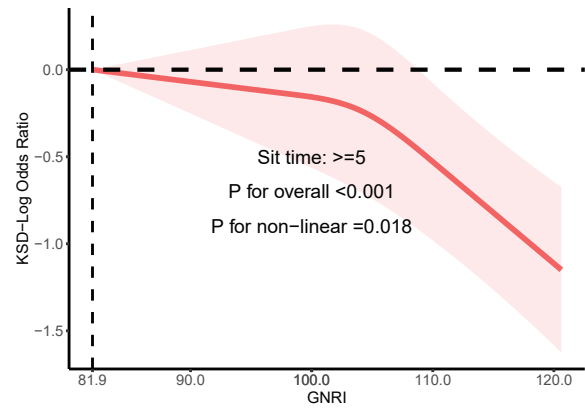

G

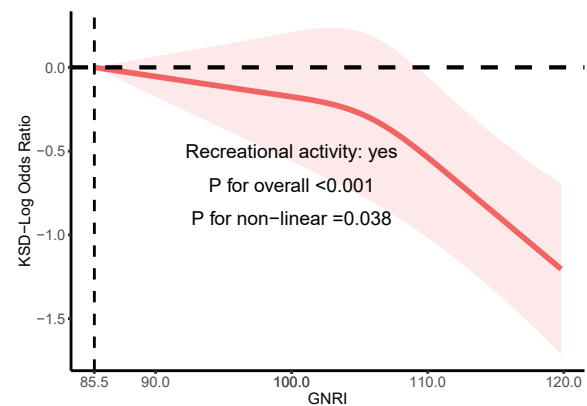

H

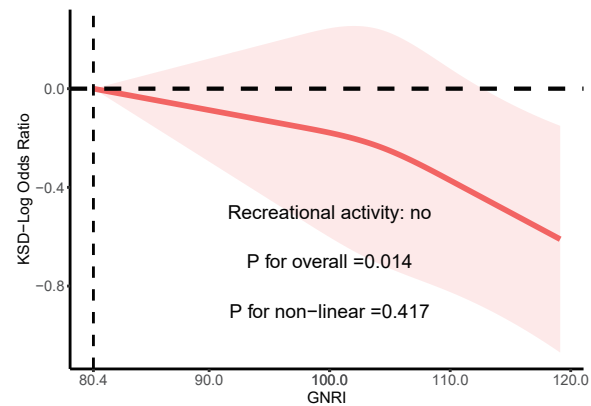

Supplement: Supplementary file 1 [file Data_Sheet_1.ZIP › Supplementary Figure3.pdf]
